# Community-based pre-pregnancy care programme improves pregnancy preparation in women with pregestational diabetes

**DOI:** 10.1007/s00125-018-4613-3

**Published:** 2018-05-09

**Authors:** Jennifer M. Yamamoto, Deborah J. F. Hughes, Mark L. Evans, Vithian Karunakaran, John D. A. Clark, Nicholas J. Morrish, Gerry A. Rayman, Peter H. Winocour, Clare Hambling, Amanda W. Harries, Michael J. Sampson, Helen R. Murphy

**Affiliations:** 1 Department of Medicine, Division of Endocrinology and Metabolism, University of Calgary, Calgary, Canada; 2 Wolfson Diabetes and Endocrine Clinic, Cambridge University Hospitals NHS Foundation Trust, Cambridge, UK; 3 Wellcome Trust–Medical Research Council Institute of Metabolic Science, University of Cambridge, Cambridge, UK; 4 North East Essex Diabetes Service (NEEDS), Colchester Hospital University NHS Foundation Trust, Colchester, UK; 5 Department of Diabetes and Endocrinology, West Suffolk Hospital NHS Trust, Bury St Edmunds, UK; 6 Department of Diabetes and Endocrinology, Bedford Hospital NHS Trust, Bedford, UK; 7 The Ipswich Diabetes Centre, Ipswich Hospitals NHS Trust, Ipswich, UK; 8 East and North Herts Institute of Diabetes and Endocrinology (ENHIDE), East and North Hertfordshire NHS Trust, Stevenage, UK; 9 Department of Public Health and Primary Care, School of Clinical Medicine, University of Cambridge, Cambridge, UK; 10 Department of Diabetes and Endocrinology, Norfolk and Norwich University Hospital, Norwich, UK; 11 Norwich Medical School, Floor 2, Bob Champion Research and Education Building, University of East Anglia, Norwich, NR4 7UQ UK

**Keywords:** Antenatal, Community-based, Diabetes, Folic acid, Glucose, Glycaemic control, Pregnancy, Pre-pregnancy care, Primary care

## Abstract

**Aims/hypothesis:**

Women with diabetes remain at increased risk of adverse pregnancy outcomes associated with poor pregnancy preparation. However, women with type 2 diabetes are less aware of and less likely to access pre-pregnancy care (PPC) compared with women with type 1 diabetes. We developed and evaluated a community-based PPC programme with the aim of improving pregnancy preparation in all women with pregestational diabetes.

**Methods:**

This was a prospective cohort study comparing pregnancy preparation measures before and during/after the PPC intervention in women with pre-existing diabetes from 1 June 2013 to 28 February 2017. The setting was 422 primary care practices and ten National Health Service specialist antenatal diabetes clinics. A multifaceted approach was taken to engage women with diabetes and community healthcare teams. This included identifying and sending PPC information leaflets to all eligible women, electronic preconception care templates, online education modules and resources, and regional meetings and educational events. Key outcomes were preconception folic acid supplementation, maternal HbA_1c_ level, use of potentially harmful medications at conception and gestational age at first presentation, before and during/after the PPC programme.

**Results:**

A total of 306 (73%) primary care practices actively participated in the PPC programme. Primary care databases were used to identify 5075 women with diabetes aged 18–45 years. PPC leaflets were provided to 4558 (89.8%) eligible women. There were 842 consecutive pregnancies in women with diabetes: 502 before and 340 during/after the PPC intervention. During/after the PPC intervention, pregnant women with type 2 diabetes were more likely to achieve target HbA_1c_ levels ≤48 mmol/mol (6.5%) (44.4% of women before vs 58.5% of women during/after PPC intervention; *p* = 0.016) and to take 5 mg folic acid daily (23.5% and 41.8%; *p* = 0.001). There was an almost threefold improvement in ‘optimal’ pregnancy preparation in women with type 2 diabetes (5.8% and 15.1%; *p* = 0.021). Women with type 1 diabetes presented for earlier antenatal care during/after PPC (54.0% vs 67.3% before 8 weeks’ gestation; *p* = 0.003) with no other changes.

**Conclusions/interpretation:**

A pragmatic community-based PPC programme was associated with clinically relevant improvements in pregnancy preparation in women with type 2 diabetes. To our knowledge, this is the first community-based PPC intervention to improve pregnancy preparation for women with type 2 diabetes.

**Data availability:**

Further details of the data collection methodology, individual clinic data and the full audit reports for healthcare professionals and service users are available from https://digital.nhs.uk/data-and-information/clinical-audits-and-registries/our-clinical-audits-and-registries/national-pregnancy-in-diabetes-audit.

**Electronic supplementary material:**

The online version of this article (10.1007/s00125-018-4613-3) contains peer-reviewed but unedited supplementary material, which is available to authorised users.

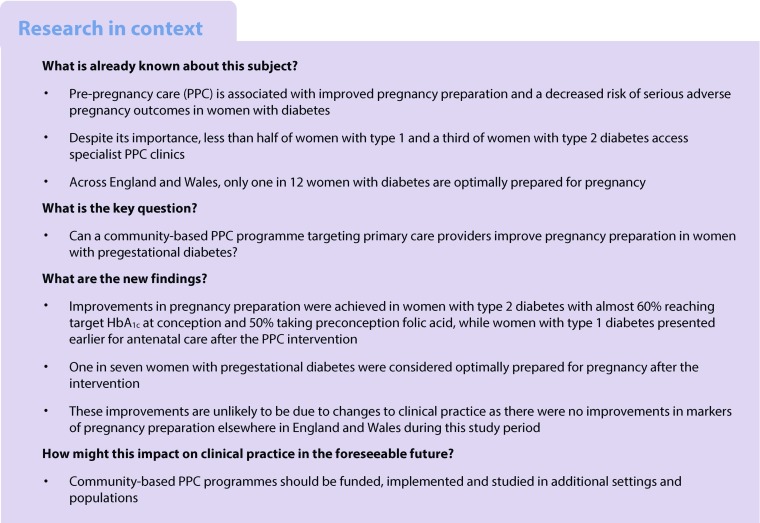



## Introduction

Women with pregestational diabetes remain at increased risk of adverse pregnancy outcomes associated with suboptimal pregnancy preparation [1, 2]. A large national cohort in the UK demonstrated that only 14% of women with type 1 diabetes and 37% of women with type 2 diabetes achieve the National Institute for Health and Care Excellence (NICE) guideline HbA_1c_ target of 48 mmol/mol (<6.5%) [3, 4]. Furthermore, only 46% and 23% of women with type 1 and type 2 diabetes respectively, were taking 5 mg of folic acid daily prior to conception [3]. This leaves much room for improvement.

Pre-pregnancy care (PPC) has been shown to improve pregnancy preparation measures such as preconception folic acid supplementation, periconception glycaemic control, avoiding potentially harmful medications and presenting for early antenatal care [5–7]. We previously demonstrated that although women with type 2 diabetes have additional obstetric risk factors (higher age, parity, BMI) compared with women who have type 1 diabetes, PPC is as effective in reducing the risk of serious adverse pregnancy outcomes in type 2 as in type 1 diabetes [5, 8]. Despite its well-established benefits and widespread recommendation, PPC attendance continues to be low [4–6, 9, 10]. Even in regions with specialist programmes, only a third of women with type 2 diabetes attend PPC [5, 6]. Conversely, the use of contraception also continues to be low, with less than half of women with type 1 and type 2 diabetes on potentially harmful prescribed medications using safe, effective methods of contraception [11].

Most women with type 2 diabetes have routine care in primary care settings, where awareness of the specific issues of diabetes pregnancy preparation is limited [8, 12]. This leads to low levels of awareness regarding the importance of safe, effective contraception to avoid an unintended pregnancy and of PPC in women who are thinking about trying for a baby. Women with type 2 diabetes are more likely to live in areas of socioeconomic deprivation and belong to ethnic minority groups so may have additional financial, cultural and ethnic barriers to accessing healthcare [8, 12].

Qualitative interviews suggested that women with type 2 diabetes had common misconceptions about their reproductive potential [13, 14], thinking it was ‘harder to conceive’ with poorly controlled diabetes and high BMI [13, 14]. Some overweight and obese women were advised that they had ‘too many risk factors’ for hormonal contraception. Others reported concerns regarding the negative views of women with type 2 diabetes and pre-pregnancy discussions [13, 15]. Information about diabetes pregnancy was seen as too ‘risk-focused’ and ‘alarming’, concentrating on ‘all the bad things that could happen’ [14]. Women stated a preference for clear practical advice about sex and diabetes with less emphasis on ‘preconception and having a baby’ [14].

There is an unmet need to improve women’s and healthcare professionals’ awareness about diabetes pregnancy risks and how they can be minimised by optimal pregnancy preparation [8]. To address this, we developed and implemented an integrated community-based PPC programme for women with pregestational diabetes (type 1, type 2 and other), focusing on engaging primary care diabetes teams. This study assesses its effectiveness on pregnancy preparation measures and pregnancy outcomes in women with pre-existing diabetes. We hypothesised that a community-based PPC programme would improve pregnancy preparation in women with type 2 diabetes.

## Methods

A multifaceted approach was taken to engage women with diabetes (type 1, type 2 and other, including MODY) as well as their primary healthcare providers, including primary care practitioners and community healthcare teams in the Eastern Academic Health Science Network (EAHSN) (see electronic supplementary material [ESM] for details of participating clinical commissioning groups [CCGs]). This included identifying and sending theoretically guided (balancing the risk of unattended pregnancy and benefits of pregnancy preparation) patient information leaflets to all eligible women (ESM Fig. 1), providing preconception care templates for use during face-to-face primary care visits (ESM Fig. 2), providing online PPC education modules and resources, as well as participating in a series of regional and local educational events for patients and healthcare professionals. The NPID patient information leaflet and consent form met the Health Research Authority requirements for clinical audit, and research ethics approval was not required.

### Pre-existing regional programme

This community-based programme was established in addition to the existing East Anglia Study Group for Improving Pregnancy Outcomes in Women with Diabetes (EASIPOD) programme [5, 8, 14]. EASIPOD was established in 2006 to improve specialist PPC clinic attendance. It involved the mailing of leaflets to women with diabetes by the specialist antenatal diabetes teams, and dissemination of information to various healthcare professionals. It was not community based and did not systematically include primary care practitioners.

### PPC leaflet

Primary care centre databases (SystmOne and EMIS) were searched using specific Read codes (https://digital.nhs.uk/article/1104/Read-Codes) to identify women with diabetes aged 16–45 years. Women who were currently pregnant, recently widowed, or who had had a previous hysterectomy, serious medical and/or psychological problems were excluded. The theoretically guided EASIPOD PPC information leaflet was revised to reflect the feedback from women who previously did not attend for PPC [14]. It was deliberately as inclusive as possible, focusing more on sex and contraception with less emphasis on ‘trying for a baby’, which previous research suggested was a potential barrier for hard-to-reach women (ESM Fig. 1). Printed and electronic copies of the revised PPC leaflet were distributed to 422 primary care centres and ten specialist diabetes maternity clinics. A nominal payment of £20 was offered to participating primary care centres to cover their administrative costs of identifying eligible women and mailing the leaflet (database search, stationery, postage).

### Electronic preconception care templates

Preconception care templates were embedded into electronic healthcare records, with pop-up alerts as an aide-memoire, to promote use during clinical encounters (ESM Fig. 2). Women not using safe, effective contraception were advised to consider a long-acting reversible contraception method. Women thinking about trying for a baby were advised to take 5 mg folic acid daily, to aim for HbA_1c_ ≤48 mmol/mol (6.5%), have a review of their current medications and their most recent renal, retinal and thyroid screening results. Specific recommendations for referral to a specialist diabetes pre-pregnancy service were made for women with HbA_1c_ >53 mmol/mol (7%), BMI>30 kg/m^2^ and women with additional medical or obstetric risk factors (ESM Fig. 2).

### Online resources

An online diabetes education programme (Cambridge Diabetes Education programme, www.cdep.org.uk; accessed 1 October 2015) was offered, free of charge, to all participating primary and specialist care practices. In addition, practices were invited to participate in a pilot evaluation of the Diabetes UK preconception information prescription www.diabetes.org.uk/professionals/resources/resources-to-improve-your-clinical-practice/information-prescriptions-qa/information-prescription--diabetes-contraception-and-pregnancy (accessed 1 May 2016).

### Regional meetings

A project midwife was supported by local project coordinators (each working 1–2 days/week) in seven CCG areas. The project coordinators directly engaged with local CCG leads, family physicians, primary care nurses, specialist diabetes teams, sexual health clinics, community groups and women with diabetes. Regional meetings were held every 4 months to review pregnancy preparation and pregnancy outcome data both during the baseline data collection before PPC programme implementation (June 2013 to September 2015; 28 months) and during/after implementation (October 2015 to February 2017; 17 months). The project midwife, local coordinators and members of the National Health Service specialist diabetes pregnancy teams attended these meetings, which allowed teams to share experiences about what PPC engagement activities worked and/or did not work.

### Project cost

The total cost for this large regional programme included non-recurring programme management and organisational costs, as well as staff who also delivered several other diabetes improvement programmes. We estimated that to deliver this model in a general UK population of 750,000 individuals including 58,000 with diabetes, the service would require recurring salary costs for a 0.25 whole-time equivalent project manager (£9,589) and three 0.25 whole-time equivalent diabetes educators / midwives (£28,767), as well as non-recurring initial modest costs for materials development (£5,000) and primary care database searches, stationery and postage (£20 × 306 = £6,120). This brings the total estimated intervention cost to £49,476 per annum.

### Data collection

Health care professionals at each specialist National Health Service maternity unit completed standardised National Pregnancy in Diabetes (NPID) web-based data entry forms (http://content.digital.nhs.uk/media/15927/NPID-Data-Collection-Form-v6/pdf/NPID_Data_Collection_Form_v6.pdf) for every pregnant woman with pregestational diabetes who delivered between 1 June 2013 and 28 February 2017. All women provided written informed consent for NPID data collection. The project midwife ensured timely data collection, validation of the data and entry into the study database. When necessary, the project coordinator contacted individual sites to ensure completeness and accuracy of the data. The pregnancy preparation and pregnancy outcome data were reviewed on a centre-by-centre basis at the quarterly regional meetings.

### Definitions and outcomes

We defined pre-existing diabetes as diabetes that had been diagnosed before pregnancy and excluded women who presented with diabetes during pregnancy. The mother’s diabetes type was added to the NPID data collection as a mandatory data item from 1 January 2015 and was obtained by linking to the most recent relevant National Diabetes Audit record. Prior to January 2015, the type of diabetes was determined by the treating clinicians and manually entered into the NPID database. Where the diabetes type entered on the NPID system was not known, National Diabetes Audit linkage was also used in order to establish a known diabetes type for as many women as possible. All types of pre-existing diabetes were included. The HbA_1c_ was measured locally, with the first and last recorded values during pregnancy collected as per the NPID audit [3]. Target HbA_1c_ was defined as ≤48 mmol/mol (6.5%) in accordance with NICE guidelines [4]. Optimal pregnancy preparation was defined as having all of the following: on 5 mg folic acid supplementation, not taking any potentially harmful medications prior to last menstrual period, first HbA_1c_ following confirmation of a positive pregnancy test ≤48 mmol/mol (6.5%) and presentation for antenatal diabetes care before 8 weeks’ gestation. Medications considered potentially harmful included secretagogues, dipeptidyl peptidase-4 (DPP-4) inhibitors, glucagon-like peptide-1 (GLP-1) agonists, thiazolidinediones, angiotensin converting enzyme inhibitors, angiotensin receptor blockers and statins.

We used standard NPID definitions for congenital anomaly, stillbirth and neonatal death and collected data on congenital anomalies for live births, stillbirths and pregnancy loss after 20 weeks’ gestation. The reported diagnoses for major congenital anomaly, as defined by the European Surveillance of Congenital Anomalies (EUROCAT), were obtained from the hospital ICD-10 codes (www.who.int/classifications/icd/en/) [16]. All congenital anomaly coding was reviewed in duplicate. Minor congenital anomalies were not included.

Infant birthweight was adjusted for maternal height, weight, infant sex and gestational age using customised centile calculators (Gestation Related Optimal Weight [GROW] centile tool v6.7.7.1 [UK] 2015 Gestation Network) (www.gestation.net/birthweight_centiles/centile_object.htm, accessed 8 November 2017).

### Statistical analysis

Differences before and during/after the implementation of the programme were analysed using *t* tests for continuous variables and Fisher’s exact test for categorical variables after ensuring test assumptions were met. A two-sided *p* value of <0.05 was considered statistically significant. Analyses were performed using Stata, version 14.1.

## Results

### Primary care participation

Out of 422 primary care centres identified, 306 (72.5%) actively participated in the programme. Collectively, they identified 5075 women with pregestational diabetes aged 16–45 years, excluded 514 for whom it was considered inappropriate, and sent the pre-pregnancy information leaflet to the remaining 4,558 (89.8%) (Table 1). The primary care response rate exceeded 75% in five out of seven CCG areas. Details according to each CCG area are shown in Table 1.

**Table 1:** Women with diabetes identified and sent PPC leaflets by CCG areas

CCG area	Primary care practices contacted	Primary care practice replies	Women with diabetes identified	PPC leaflets sent
Norfolk and Waveney^a^	110	83 (75.5%)	1438	1246
Cambridgeshire and Peterborough	107	81 (75.7%)	1289	1276
East and North Hertfordshire	59	46 (78%)	699	583
Ipswich and East Suffolk	40	35 (87.5%)	638	549
West Suffolk	25	24 (96.0%)	425	397
Bedfordshire	40	13 (32.5%)	199	176
North East Essex	41	24 (58.5%)	387	331
Total	422	306 (72.5%)	5075	4558 (89.8%)

^a^Norfolk and Waveney are comprised of the following CCGs: Norwich, North Norfolk, South Norfolk, West Norfolk, Great Yarmouth and Waveney

Login codes for the online diabetes education programme (Cambridge Diabetes Education Programme) were created for up to 1500 users. Of those, 311 individuals registered for the programme. This included individuals from primary care practices (182), hospitals (57) and other institutions such as care homes, pharmacies, community trusts etc. (72). A total of 221 individuals started at least one module and 75 completed at least one module during the 17 month PPC programme implementation phase. The most popular completed modules were: What is diabetes? (*n* = 63), Hypoglycaemia (*n* = 52), Hyperglycaemia (*n* = 39), Screening and early detection of type 2 diabetes (*n* = 36) and Preconception care (*n* = 32).

### Specialist antenatal diabetes care participation

Of the 842 women who presented with pregnancies complicated by diabetes, 513 (60.9%) had type 1 diabetes, 318 (37.8%) had type 2 diabetes and 11 (1.3%) had other types of diabetes in pregnancy. Specialist clinics varied by number of pregnancies in women with pre-existing diabetes, which ranged from 32–173 women. A total of 502 women who attended before and 340 women who attended during/after the PPC programme were included.

### Maternal characteristics

The proportion of women with type 1 and type 2 diabetes remained consistent over time (61.4% before vs 60.3% with type 1 diabetes during/after the programme; *p* = 0.77), as did maternal BMI at booking (29.3 ± 7.2 kg/m^2^ vs 29.8 ± 6.9 kg/m^2^; *p* = 0.29) (ESM Table 1). Women who attended during/after the implementation of the PPC programme were younger (31.4 ± 5.9 vs 32.5 ± 5.9 years; *p* = 0.0074). Details of maternal characteristics before and during/after intervention are found in ESM Table 1.

Less than half of women with type 1 diabetes (41.0% before and 36.1% during/after PPC; *p* = 0.27) and approximately one in ten with type 2 diabetes (12.4% before and 9.9% during/after PPC; *p* = 0.59) had a BMI in the normal range of <24.9 kg/m^2^. Details of maternal characteristics by type of diabetes (type 1 or type 2) are shown in Table 2.

**Table 2:** Maternal characteristics before and during/after implementation of a regional PPC programme by type of diabetes

	Type 1 diabetes			Type 2 diabetes		
Characteristic	Before PPC	During/after PPC	*p* value	Before PPC	During/after PPC	*p* value
	n=308	n=205		n=186	n=132	
Age in years at delivery, mean (SD)	31.2 (5.9)	30.2 (5.8)	0.058	34.7 (5.2)	33.4 (5.4)	0.026
Diabetes treatment at first visit, *n* (%)						
Insulin pump	71 (23.1)	46 (22.4)	0.92	0 (0)	0 (0)	–
Metformin	23 (7.5)	22 (10.7)	0.21	134 (72.0)	92 (69.7)	0.71
Weight at booking in kg, mean (SD)^a^	71.8 (13.8)	74.2 (15.2)	0.068	91.2 (24.5)	90.8 (21.5)	0.87
BMI at booking in kg/m^2^, mean (SD)	26.5 (4.6)	27.3 (5.3)	0.068	34.3 (8.0)	33.9 (7.2)	0.71
Normal (<24.9)	126 (41.0)	74 (36.1)	0.27	23 (12.4)	13 (9.9)	0.59
Overweight (25–29.9)	125 (40.7)	75 (36.6)	0.36	31 (16.7)	28 (21.2)	0.31
Obese (≥30)	56 (18.2)	56 (27.3)	0.017	132 (71.0)	91 (68.9)	0.71

^a^Maternal weight at booking was available for all but one participant with type 1 diabetes before PPC

For women with type 1 diabetes, 23.1% and 22.4% used insulin pump therapy before and during/after PPC respectively, and 7.5% and 10.7% were taking metformin in early pregnancy before and during/after PPC respectively. For women with type 2 diabetes, 72.0% and 69.7% respectively were taking metformin in early pregnancy.

### Pregnancy preparation

Almost two-thirds (64.2–64.6%) of women with type 1 diabetes were on folic acid before conception with very few (<4%) taking any potentially harmful medications before and during/after PPC implementation (Table 3). In women with type 1 diabetes, only gestational age at booking (8.4 ± 3.5 vs 7.6 ± 3.7 weeks; *p* = 0.020) and booking prior to 8 weeks’ gestation (54% vs 67.3%; *p* = 0.003) improved significantly (Table 3). There was a trend towards increasing ‘optimal’ preparation for pregnancy, but this did not reach statistical significance (10.6% vs 16.3%; *p* = 0.086).

**Table 3:** Measures of pregnancy preparation before and during/after implementation of a regional PPC programme by type of diabetes

	Type 1 diabetes			Type 2 diabetes		
Measure	Before PPC	During/after PPC	*p* value	Before PPC	During/after PPC	*p* value
	n=308^a^	n=205^a^		n=186^a^	n=132^a^	
Booking time	*n* = 304	*n* = 205		*n* = 185	*n* = 132	
Gestational age at booking in weeks, mean (SD)	8.4 (3.5)	7.6 (3.7)	0.020	10.5 (4.5)	9.8 (4.9)	0.25
Booking prior to 8 weeks, *n* (%)	164 (54.0)	138 (67.3)	0.003	62 (33.5)	53 (40.2)	0.24
HbA_1c_	*n* = 287	*n* = 198		*n* = 178	*n* = 130	
HbA_1c_ at first contact in mmol/mol, mean (SD)	62.2 (18.3)	61.5 (17.3)	0.65	52.2 (14.9)	50.4 (14.8)	0.30
HbA_1c_ at first contact in %, mean (SD)	7.8 (3.8)	7.8 (3.7)	0.65	6.9 (3.5)	6.8 (3.5)	0.30
First HbA_1c_ ≤48mmol/mol, *n* (%)	62 (21.6)	46 (23.2)	0.74	79 (44.4)	76 (58.5)	0.016
Preconception folic acid	*n* = 288	*n* = 195		*n* = 164	*n* = 123	
Preconception folic acid any dose, *n* (%)	185 (64.2)	126 (64.6)	1.00	60 (36.6)	60 (48.9)	0.041
Preconception folic acid 5 mg dose daily^b^, *n* (%)	174 (60.6)	113 (58.0)	0.57	38 (23.5)	51 (41.8)	0.001
Potentially harmful medication	*n* = 274	*n* = 204		*n* = 169	*n* = 131	
On at least one potentially harmful medication^c^, *n* (%)	10 (3.7)	3 (1.5)	0.17	27 (16.0)	16 (12.2)	0.41
Two or more potentially harmful medications, *n* (%)	0 (0)	0 (0)	–	7 (4.1)	2 (1.5)	0.31
Optimal pregnancy preparation^d^			0.086			0.021
*n*	*n* = 236	*n* = 190		*n* = 138	*n* = 119	
*n* (%)	25 (10.6)	31 (16.3)		8 (5.8)	18 (15.1)	

^a^*n* values are shown below for the number of participants for which these data were available for each category of measures or each individual measure

^b^*n* values for numbers of participants with data for 5 mg daily dose of folic acid are 287 (type 1 diabetes before PPC); 195 (type 1 diabetes during/after PPC); 162 (type 2 diabetes before PPC); 122 (type 2 diabetes during/after PPC)

^c^Potentially harmful medications include secretagogues, dipeptidyl peptidase-4 (DPP-4) inhibitors, glucagon-like peptide-1 (GLP-1) agonists, thiazolidinedione, angiotensin converting enzyme inhibitor / angiotensin receptor blockers and statin

^d^Optimal pregnancy preparation defined as first HbA_1c_ ≤48 mmol/mol (6.5%), on folic acid 5mg daily prior to last menstrual period, booking at ≤8 weeks gestation and no harmful medications prior to last menstrual period

In contrast, women with type 2 diabetes were significantly more likely to achieve a target HbA_1c_ ≤48mmol/mol (6.5%) (44.4% before vs 58.5% during/after PPC implementation; *p* = 0.016) and to take preconception folic acid, both for ‘any dose’ (36.6% before vs 48.9% during/after PPC implementation; *p* = 0.041) and for the NICE recommended 5 mg dose (23.5% before and 41.8% during/after PPC implementation; *p* = 0.001). There was almost a threefold improvement in ‘optimal’ pregnancy preparation during/after PPC implementation in women with type 2 diabetes (5.8% before and 15.1% during/after PPC implementation; *p* = 0.021) (Table 3). Overall, 16% of women with pregestational diabetes were considered ‘optimally’ prepared for pregnancy during/after implementation (ESM Table 2). There was no change in the proportion of women with type 2 diabetes taking potentially harmful medications (16% before and 12.2% during/after PPC implementation; *p* = 0.41).

### Pregnancy outcomes

Pregnancy outcomes for the 812 singleton pregnancies of women with type 1 and type 2 diabetes are shown in Table 4. There were five perinatal deaths in type 1 diabetes pregnancies before (three stillbirths and two neonatal deaths) and none during/after PPC implementation. However, there were more congenital anomalies in type 1 diabetes offspring during/after PPC implementation, meaning that the overall rate of serious adverse pregnancy outcome was unchanged.

**Table 4:** Pregnancy outcomes before and during/after implementation of PPC programme by type of diabetes

	Type 1 diabetes			Type 2 diabetes		
	Before PPC	During/after PPC	*p* value	Before PPC	During/after PPC	*p* value
	n=302^a^	n=202^a^		n=183^a^	n=131^a^	
Pregnancy outcome^b^	*n* = 302	*n* = 202		*n* = 183	*n* = 131	
Live birth, *n* (%)	278 (92.1)	187 (92.6)	0.87	172 (94.0)	123 (93.9)	1.00
Miscarriage, *n* (%)	18 (6.0)	14 (6.9)	0.71	8 (4.4)	5 (3.8)	1.00
Termination, *n* (%)	3 (1.0)	1 (0.5)	0.65	2 (1.1)	0 (0)	0.51
Delivery^c^	*n* = 277	*n* = 187		*n* = 169	*n* = 126	
Gestational age at delivery, mean (SD)	36.8 (2.2)	36.9 (1.7)	0.90	37.4 (1.9)	37.3 (1.75)	0.49
Prematurity						
<37 weeks, *n* (%)	98 (35.4)	79 (42.3)	0.15	35 (20.7)	35 (27.8)	0.17
<34 weeks, *n* (%)	26 (9.4)	12 (6.4)	0.30	8 (4.7)	4 (3.2)	0.57
Birthweight (g)			0.43			0.85
*n*	*n* = 280	*n* = 185		*n* = 173	*n* = 126	
mean (SD)	3379.7 (700.0)	3429.6 (618.5)		3235.2 (665.5)	3249.7 (655.1)	
Infant birth centiles^d^	*n* = 275	*n* = 185		*n* = 169	*n* = 126	
Large for gestational age, *n* (%)	121 (44.0)	86 (46.5)	0.63	32 (18.9)	28 (22.2)	0.56
Extremely large for gestational age, *n* (%)	80 (29.1)	59 (31.9)	0.54	17 (10.1)	14 (11.1)	0.84
Small for gestational age, *n* (%)	15 (5.5)	8 (4.3)	0.67	29 (17.2)	14 (11.1)	0.18
Advanced neonatal care			0.037			0.21
*n*	*n* = 277	*n* = 187		*n* = 172	*n* = 123	
*n* (%)	129 (46.6)	106 (56.7)		53 (30.8)	47 (38.2)	
Other pregnancy outcomes						
Congenital malformation			0.084			0.72
*n*	*n* = 267	*n* = 177		*n* = 168	*n* = 121	
*n* (%)	10 (3.8)	14 (7.9)		4 (2.4)	4 (3.3)	
Stillbirth			0.28			0.31
*n*	*n* = 281	*n* = 187		*n* = 173	*n* = 126	
*n* (%)	3 (1.1)	0 (0)		1 (0.6)	3 (2.4)	
Neonatal death			0.52			1.00
*n*	*n* = 269	*n* = 176		*n* = 166	*n* = 112	
*n* (%)	2 (0.74)	0 (0)		1 (0.6)	0 (0)	
Perinatal mortality			0.16			0.40
*n*	*n* = 272	*n* = 176		*n* = 167	*n* = 115	
*n* (%)	5 (1.8)	0 (0)		2 (1.2)	3 (2.6)	
	*n* = 259	*n* = 171		*n* = 163	*n* = 113	
Serious adverse outcome^e^, *n* (%)	14 (5.4)	14 (8.2)	0.32	6 (3.7)	7 (6.2)	0.39

^a^*n* values are shown for the number of participants for which these data were available for each category of measures or each individual measure

^b^Reported on all singleton pregnancies

^c^Reported on live and stillbirths

^d^Large for gestational age >90th centile, extremely large for gestational age >97.7th centile, small for gestational age <10th centile as per GROW customised centiles

^e^Serious adverse outcome: malformation with or without termination of pregnancy, stillbirth or neonatal death

There were no differences in most perinatal morbidity outcomes before and during/after PPC implementation. There was evidence for an overall increase in neonatal intensive care unit admissions (40.4% before vs 48.9% during/after PPC implementation; *p* = 0.022), which is likely due to a trend towards earlier obstetric intervention (29.7% vs 36.1% preterm delivery <37 weeks gestation; *p* = 0.071) (ESM Table 3). This was driven mainly by a higher proportion of type 1 diabetes neonates requiring advanced neonatal care (46.6% before vs 56.7% during/after PPC implementation; *p* = 0.022) (Table 4).

## Discussion

We report on a pragmatic community-based PPC programme that is simple and effective in improving pregnancy preparation in women with type 2 diabetes. Almost 75% of primary care centres were actively engaged in identifying women with diabetes and sending them information about sex, contraception and pregnancy. Significant improvements in pregnancy preparation were seen in women with type 2 diabetes with almost 60% reaching target HbA_1c_ at conception, and 50% taking preconception folic acid. Although most women (85%) with type 2 diabetes were not optimally prepared for pregnancy, there was an overall threefold improvement in pregnancy preparation measures. Women with type 1 diabetes had higher than average rates of folic acid supplementation before and during/after with the only significant difference being earlier presentation for antenatal care during and after implementation of the PPC programme.

This community-based PPC programme was developed to address the limitations of a previous regional programme which was effective for improving pregnancy outcomes but was focused on specialist pre-pregnancy clinics and did not adequately engage with primary care teams [5]. With a 90% increase in type 2 diabetes over 15 years and increasing numbers of younger women with type 2 diabetes, primary care practitioners (nurses and family physicians) are increasingly providing routine diabetes care [17]. This programme was primarily focused on raising awareness about the importance of safe, effective contraception and/or PPC among primary care teams.

Measures such as improvement in folic acid preparation associated with the previous PPC programme have been maintained over the decade since it was first introduced [5]. Specifically, 46% and 33% of women with type 1 and type 2 diabetes were on preconception folic acid between 2006 and 2009, compared with 64% and 37% respectively prior to the current intervention [5]. Thus, there had been ongoing improvements over time in type 1 diabetes but little or no change in type 2 diabetes pregnancy. This programme demonstrated clinically relevant improvements in type 2 diabetes pregnancy with 49% of women with type 2 diabetes now taking preconception folic acid.

Using the same definitions and data collection procedures allows us to directly compare our findings with the NPID audit of women with pregestational diabetes in England and Wales. Only 15% and 38% of women with type 1 and type 2 diabetes in NPID achieved first trimester HbA_1c_ <48mmol/mol (6.5%) [18]. In our study, 23% and 58% of women with type 1 and type 2 diabetes, respectively, achieved NICE guideline HbA_1c_ targets during the PPC programme. Similar improvements were seen with 5 mg daily folic acid supplementation, with 42% and 23% of women with type 1 and type 2 diabetes in NPID, compared with 58% and 42%, respectively, during our study. This is almost double the national rates for pre-pregnancy folic acid supplementation in type 2 diabetes pregnancy. Finally, only one in 12 women nationally were considered well prepared for pregnancy (defined as HbA_1c_ <48mmol/mol (6.5%), 5 mg folic acid daily and no harmful medications). In our study, one in seven women with pre-existing diabetes were considered optimally prepared for pregnancy, which included a more stringent criterion of booking before 8 weeks’ gestation.

In our study, more substantial improvements in pregnancy preparation were observed in type 2 diabetes compared with type 1 diabetes. There may be many reasons for this difference. First, our programme targeted primary care practitioners and community healthcare teams, which would have had a bigger impact on women with type 2 diabetes who are more likely to receive their care in a community setting [8]. Second, women with type 2 diabetes tend to have a shorter duration of diabetes and a less severe glycaemic disturbance. They can be treated more effectively with oral agents and are more likely to reach target HbA_1c_ than women with type 1 diabetes. Finally, women with type 2 diabetes are more likely to be prescribed medications for hypertension and lipid lowering, offering an achievable target for improvement in overall pregnancy preparation [8].

In contrast, in women with type 1 diabetes, the rates of folic acid supplementation and early presentation for antenatal care compared favourably to national data, but optimising glycaemic control remains a major hurdle. The recent randomised controlled trial of continuous glucose monitoring (CONCEPTT) highlighted that even with high rates of continuous glucose monitoring and insulin pump use, only about 50% of women attending specialist PPC clinics were able to attain target HbA_1c_ levels [19]. It also described a high proportion (60%) of women with type 1 diabetes who were overweight and obese before pregnancy. Our data suggested an increase in body weight and maternal BMI, even over the course of the programme, with an average 2.4 kg and 0.8 BMI point increase, even though women were entering pregnancy at a younger age. Interestingly, there were no such changes in women with type 2 diabetes. There is an unmet need to develop evidence-based dietary advice and weight-management guidelines for women with type 1 and type 2 diabetes.

While overall pregnancy preparation improved after the implementation of our programme, 84% of women with pregestational diabetes are still not ‘optimally’ prepared for pregnancy. This highlights that much more needs to be done. If the programme had been continued for a longer period, more women may have benefitted. Ideally it would be implemented over at least 12–24 months, to allow for maximum participation before assessing its impact. Improvements in the use of safe, effective contraception and in folic acid supplementation for women not using appropriate contraception should be prioritised in primary care. Also, further improvements would be achieved if women with type 2 diabetes and healthcare teams were more aware of the importance of immediate referral for antenatal care following confirmation of pregnancy. In specialist care, more attention is required in helping women optimise glycaemic control.

Our study has some important strengths. We describe an intervention that was simple, inexpensive, sustainable and easily reproducible in other regions. It was performed over a short time (only 17 months), with no substantial changes to clinical practice guidelines and/or diabetes technology use and no documented changes in national pregnancy preparation as recorded by the same NPID measures from 2014 to 2016 [18]. Thus, the improvements seen with the initiation of our programme are unlikely related to wider changes in the care of women with diabetes. We were able to demonstrate a benefit of this programme despite the existing regional programme and above average baseline measures of pregnancy preparation compared with NPID. Finally, it was simple to establish and was achieved at a very modest cost of less than £50,000 per year. The 2014 National Reference Costs for the lifetime specialist health care costs for each congenital anomaly were estimated as £668,098 for neural tube defects, £434,340 for cardiac malformations, £82,972 for gastrointestinal defects and £47,160 for cleft lip and palate (personal communication, P. King, Royal Derby Hospitals NHS Foundation Trust, Derby, UK), suggesting that prevention of one cleft lip and palate would cover the programme costs.

However, our study also has limitations. Because our programme was multifaceted, we are unable to comment on which component of the programme was most effective. We hypothesise that the systematic provision of information to all women with diabetes, the face-to-face contact with primary care teams and the electronic preconception care templates are all relevant. The uptake of other aspects such as the specific preconception care module of the online education programme was disappointing. Our study was likely not large enough or of long-enough duration to detect differences in pregnancy outcomes which would have required 580 pregnancies in the follow-up period to detect 30% reduction in serious adverse outcomes [5]. Unfortunately, the limited funding arrangements did not allow a longer duration of follow up. Because we compare the programme before and during/after, we are unable to comment on overall rates of PPC attendance and their relation to pregnancy outcomes. However, this is a pragmatic approach to assessing the programme’s effectiveness. Furthermore, we are not confounded by potential differences in women who may and may not seek PPC. We lack information on other important confounders including diabetes duration, smoking and social disadvantage.

PPC remains an area of diabetes care in which measurable improvements are achievable. We must continue to develop and implement strategies such as electronic preconception care templates that improve the uptake of safe, effective methods of contraception and/or access to PPC for all women with diabetes. The suboptimal glycaemic control and rising rates of obesity in type 1 diabetes also require attention. These types of programmes should be funded, implemented and studied in additional settings and populations.

## Electronic supplementary material

ESM (PDF 831 kb)

## Data Availability

Further details of the data collection methodology, individual clinic data and the full audit reports for healthcare professionals and service users are available from https://digital.nhs.uk/data-and-information/clinical-audits-and-registries/our-clinical-audits-and-registries/national-pregnancy-in-diabetes-audit.

## Abbreviations

CCG: Clinical commissioning group
EAHSN: Eastern Academic Health Science Network
EASIPOD: East Anglia Study Group for Improving Pregnancy Outcomes in Women with Diabetes
GROW: Gestation Related Optimal Weight
NICE: National Institute for Health and Care Excellence
NPID: National Pregnancy in Diabetes
PPC: Pre-pregnancy care
