# Supplementary material for: Community-based pre-pregnancy care programme improves pregnancy preparation in women with pregestational diabetes
Source: Diabetologia. 2018 May 9;61(7):1528–37. doi: 10.1007/s00125-018-4613-3 (PMC6445478; doi:10.1007/s00125-018-4613-3)
Supplement: Supplementary file 1 — (PDF 831 kb) [file 125_2018_4613_MOESM1_ESM.pdf]

Electronic Supplementary Material

Summary of organisations participating in the EAHSN pre-pregnancy care programme

ESM Table 1: Maternal characteristics before and during/after implementation of pre-pregnancy care program

ESM Table 2: Pregnancy preparation before and during/after implementation of pre-pregnancy care program

ESM Table 3: Pregnancy outcomes before and during/after implementation of pre-pregnancy care program

ESM Figure 1: Pre-pregnancy care leaflet

ESM Figure 2: Electronic preconception care template

Summary of organisations participating in the EAHSN prepregnancy care programme

<u>CCGs covered by EAHSN Diabetes Programme</u>

NHS Norwich CCG
Room 202
City Hall
St Peters Street
Norwich
NR2 1NH

NHS North Norfolk CCG
1 Mill Close
Aylsham
NR11 6LZ

NHS South Norfolk CCG
Lakeside 400
Old Chapel Way
Broadland Business Park
Thorpe St Andrew
Norwich
Norfolk
NR7 0WG

NHS West Norfolk CCG
Kings Court
Chapel Street
Kings Lynn
PE30 1EL

NHS Great Yarmouth and Waveney CCG
1 Common Lane North
Beccles
NR34 9BN

NHS Cambridgeshire and Peterborough CCG
Lockton House
Clarendon Road
Cambridge
CB2 8FH

NHS East and North Hertfordshire CCG
Charter House
Parkway
Welwyn Garden City
Herts
AL8 6JL

NHS Ipswich and East Suffolk CCG
Rushbrook House
Paper Mill Lane
Bramford
Ipswich
IP8 4DE

NHS North East Essex CCG
Aspen House
Stephenson Road
Severalls Business Park
Colchester CO4 9QR

NHS Bedfordshire CCG
Capability House
Silsoe
Bedfordshire
MK45 4HR

NHS West Suffolk CCG
West Suffolk House
Western Way
Bury St Edmunds
Suffolk
IP33 3YU

Acute Trusts covered by EAHSN Diabetes Programme

Norfolk & Norwich University Hospitals NHS Foundation Trust
Colney Lane
Norwich
NR4 7UY

James Paget University Hospitals NHS Foundation Trust
Lowestoft Road
Gorleston
Great Yarmouth
Norfolk & Norwich University NHS Foundation Trust NR31 6L

The Queen Elizabeth Hospital, Kings Lynn, NHS Foundation Trust
Gayton Road
Kings Lynn
PE30 4ET

Cambridge University Hospitals NHS Foundation Trust
Cambridge Biomedical Campus
Hills Road
Cambridge
CB2 0QQ

Hinchingbrooke Healthcare NHS Trust
Hinchingbrooke Hospital
Hinchingbrooke Park
Huntingdon
Cambridgeshire
PE29 6NT

East & North Hertfordshire NHS Trust
Lister Hospital
Coreys Mill Lane
Stevenage
SG1 4AB

Ipswich Hospital NHS Trust
Heath Road
Ipswich
IP4 5PD

Colchester Hospital University NHS Foundation Trust
Colchester General Hospital
Trust Offices
Main Building
Turner Road
Colchester
Essex
CO4 5JL

Bedford Hospital NHS Trust
South Wing
Kempston Road
Bedford
MK41 9DJ

West Suffolk Hospital NHS Foundation Trust
Hardwick Lane
Bury St Edmunds
Suffolk
IP33 2QZ

ESM Table 1: Maternal characteristics before and during/after implementation of pre-pregnancy care (PPC) programme

	Before PPC n=502	During/after PPC n=340	*p*-value
Age in years at delivery – Mean (SD)	32.5 (5.9)	31.4 (5.9)	0.0074
Type of Diabetes – n (%)			
Type 1 diabetes	308 (61.4)	205 (60.3)	0.77
Type 2 diabetes	186 (37.1)	132 (38.8)	0.61
Other	8 (1.6)	3 (0.9)	0.54
Diabetes treatment at first visit – n (%)			
Insulin	348 (69.3)	245 (71.2)	0.40
Metformin	163 (32.5)	116 (34.1)	0.66
Secretagogue	11 (2.19)	3 (0.88)	0.18
DPP-4 Inhibitor	4 (0.8)	1 (0.29)	0.65
GLP-1 Inhibitor	2 (0.4)	2 (0.6)	1.00
Thiazolidinediones	1 (0.2)	1(0.3)	1.00
	n=501*	n=340	
Weight at booking in Kg – Mean (SD)	78.9 (20.8)	80.6 (19.6)	0.24
BMI at booking in kg/m^2 – Mean (SD)	29.3 (7.2)	29.8 (6.9)	0.29
Normal (<24.9) – n (%)	155 (30.9)	88 (25.9)	0.12
Overweight (25-29.9) – n (%)	158 (31.5)	105 (30.9)	0.88
Obese (≥30) – n (%)	188 (37.5)	147 (39.8)	0.099
	n=285[a]	n=206	
Documented retinopathy in the first trimester – n (%)	130 (45.6)	89 (43.2)	0.65
	n=270	n=201	
Documented maculopathy in the first trimester – n (%)	31 (11.5)	17 (8.46)	0.36
	n=258	n=181	
Documented retinopathy in the last trimester – n (%)	130 (50.4)	80 (44.2)	0.21
	n=252	n=174	
Documented maculopathy in the last trimester – n (%)	24 (9.5)	9 (5.2)	0.14
	n=452	n=339	
Documented ischemic heart disease – n (%)	1 (0.2)	2(0.6)	0.58
	n=454	n=340	
Treated hypertension – n (%)	20 (4.4)	24 (7.1)	0.12

*These n values refer to the number of participants for which these data were available for each measure.
[a] There were substantial missing data for retinopathy measures because retinal screening data items are poorly captured in National Pregnancy in Diabetes (NPID).

ESM Table 2: Measures of pregnancy preparation before and during/after implementation of pre-pregnancy care programme (PPC)

	Before PPC<br>n=502	During/after PPC<br>n=340	*p*-value
	n=496	n=340	
Gestational age at booking in weeks – mean (SD)	9.2 (4.0)	8.5 (4.3)	0.021
Booking prior to 8 weeks – n (%)	229 (46.2)	193 (56.8)	0.003
	n=472*	n=331	
HbA_{1c} at first contact in mmol/mol – mean (SD)	58.1 (17.7)	56.9 (17.2)	0.34
HbA_{1c} at first contact in % – mean (SD)	7.5 (3.8)	7.4 (3.7)	0.34
HbA_{1c} at target (≤48 mmol/mol) – n (%)	148 (31.4)	124 (37.5)	0.081
	n=459	n=321	
Folic acid any dose – n (%)	250 (54.5)	189 (58.9)	0.24
	n=456	n=320	
Folic acid dose of 5mg dose – n (%)	217 (47.6)	166 (51.9)	0.24
	n=451	n=338	
On at least one harmful medication[a] – n (%)	37 (8.2)	19 (5.6)	0.21
On 2 or more harmful medications – n (%)	7 (1.6)	2 (0.6)	0.31
	n=379	n=312	
Optimal pregnancy preparation[b] – n (%)	36 (9.5)	50 (16.0)	0.011

*These n values refer to the number of participants for which these data were available for each measure.

[a] Harmful medications include: secretagogues, dipeptidyl peptidase-4 (DPP-4) inhibitors, glucagon-like peptide-1 (GLP-1) agonists, thiazolidinedione, angiotensin converting enzyme inhibitor/angiotensin receptor blockers, and statin

[b] Optimal pregnancy preparation defined as: first HbA_{1c} ≤48 mmol/mol, on folic acid 5mg prior to last menstrual period, booking at ≤8 weeks gestation, and no harmful medications prior to last menstrual period

ESM Table 3: Pregnancy outcomes before and during/after implementation of pre-pregnancy care programme (PPC)

	Before PPC n=493	During/After PPC n=336	*p*-value
Pregnancy outcome [a]			
Live birth – n (%)	457 (92.7)	313 (93.2)	0.89
Miscarriage – n (%)	27 (5.5)	19 (5.7)	1.00
Termination – n (%)	5 (1.0)	1 (0.3)	0.41
Delivery [b]	n=452*	n=316	
Gestational age at delivery – Mean (SD)	37.1 (2.1)	37.0 (1.7)	0.78
Prematurity			
<37 weeks – n (%)	134 (29.7)	114 (36.1)	0.071
<34 weeks – n (%)	34 (7.5)	16 (5.1)	0.19
	n=460	n=314	
Birthweight (g) – Mean (SD)	3233.7 (685.6)	3347.0 (636.3)	0.48
Infant birth centiles [c]	n=450	n=314	
Large for gestational age – n (%)	154 (34.2)	115 (36.6)	0.54
Extremely large for gestational age – n (%)	97 (21.6)	73 (23.3)	0.60
Small for gestational age – n (%)	45 (10.0)	22 (7.0)	0.16
	n=456	n=313	
Advanced neonatal care – n (%)	184 (40.4)	153 (48.9)	0.022
Other pregnancy outcomes	n=441	n=301	
Congenital malformation – n (%)	15 (3.4)	18 (6.0)	0.10
	n=461	n=316	
Stillbirth – n (%)	4 (0.9)	3 (1.0)	1.00
	n=442	n=290	
Neonatal death – n (%)	3 (0.7)	0 (0)	0.28
	n=446	n=293	
Perinatal mortality – n (%)	7 (1.6)	3 (1.0)	0.75
	n=428	n=286	
Serious adverse outcome – n (%)	21 (4.9)	21 (7.3)	0.20

*These n values refer to the number of participants for which these data were available for each measure.

[a] These outcomes are applicable only for singleton pregnancies. There was a total of 13 twin gestations (9 (1.8%) pre-PPC and 4 (1.2%) post-PPC. No twin pregnancy ended in stillbirth or neonatal death.

[b] Reported on live and stillbirths

[c] Large for gestational age >90th centile, extremely large for gestational age >97.7th centile, small for gestational age <10th centile as per GROW customised centiles

[d] Serious adverse outcome: malformation with or without termination of pregnancy, stillbirth, or neonatal death

ESM Figure 1: Pre-pregnancy care leaflet

Already pregnant? We can help.

If you are pregnant and you didn't plan your pregnancy we can help you. The specialist team at the hospital will see you as soon as possible to give you support, information and care.

This is what you need to do

1. As soon as you can, contact your GP/diabetes team. They will assist with:
   - A referral to the maternity unit. You can expect an appointment within 1-2 weeks
   - Getting a prescription for Folic Acid. You may need a 5mg dose and this is only available by prescription
   - A review of your medications
2. Start testing your blood glucose levels before meals, 1 hour after meals, and before bed so that your diabetes treatment can be safely adjusted.

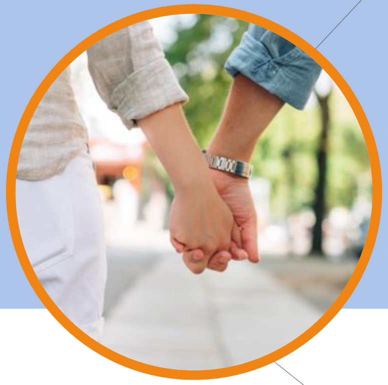

Remember...

Your diabetes team are here to help you!

We can help you with contraception, planning your pregnancy and help you to have a healthy baby. If you have any questions about contraception or pregnancy get in contact today.

The Eastern Academic Health Science Network

eahsn.org.uk is working with local healthcare teams to support women with diabetes who are planning pregnancy.
Email: EAHSN.diabetes@nhs.net

Useful resources/links

fpa.org.uk The website of the Family Planning Association – all you ever wanted to know about contraceptive methods, plus an easy-to-use tool to find the best contraceptive methods for you.

diabetes.org.uk Good section on pregnancy in the "Living with diabetes" section, on contraception in the "teenagers" section, and giving up smoking advice.

womenwithdiabetes.net Lots of information, video clips and other women's stories.

gofolic.org.uk Information on the benefits of folic acid and the Before You Frolic, Go Folic! Campaign.

Pregnant with diabetes app Free from Google play. Loads of information including planning for pregnancy

If this information has come at a bad time, causes you concern or is not relevant to you, please accept our sincere apologies and feel free to let us know.

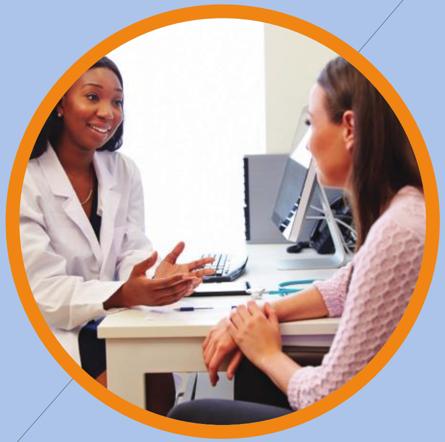

Sex, contraception and pregnancy

Important information for women with Type 1 and Type 2 diabetes

eahsn
Eastern Academic
Health Science Network

Why a bit of planning is important

If you have Type 1 or Type 2 diabetes and are having sex it is important to think about contraception and pregnancy.

Contraception

If you are having sex and are not planning on having a baby soon, talk to your GP about the safest and best contraception for you. Your diabetes team can help you with more information also.

Thinking about getting pregnant

Most women with diabetes have normal pregnancies resulting in healthy babies. However, having diabetes can increase the chance of serious complications both for you and your baby. We also know the first 6 weeks of pregnancy is the most crucial for your baby's development. If you are thinking about having a baby, planning for pregnancy and looking after yourself is important for you and your baby's health. Try and see your GP/ practice nurse or the diabetes team as soon as you start thinking about having a baby. The earlier the better, and even 6-12 months before stopping contraception is a great time to start the discussion.

Risks and complications

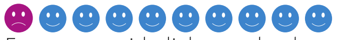

For women with diabetes who do not plan their pregnancy, the risk of a serious complication (e.g. stillbirth, serious heart or birth defect) is about 1 in 10.

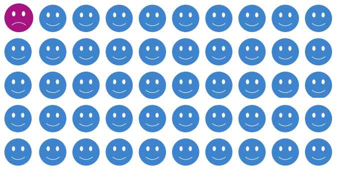

Reassuringly, if you do plan your pregnancy with your diabetes team, your risk of serious complications falls closer to that of women without diabetes (1 in 50).

The best advice we can give you is to try not to leave things to chance

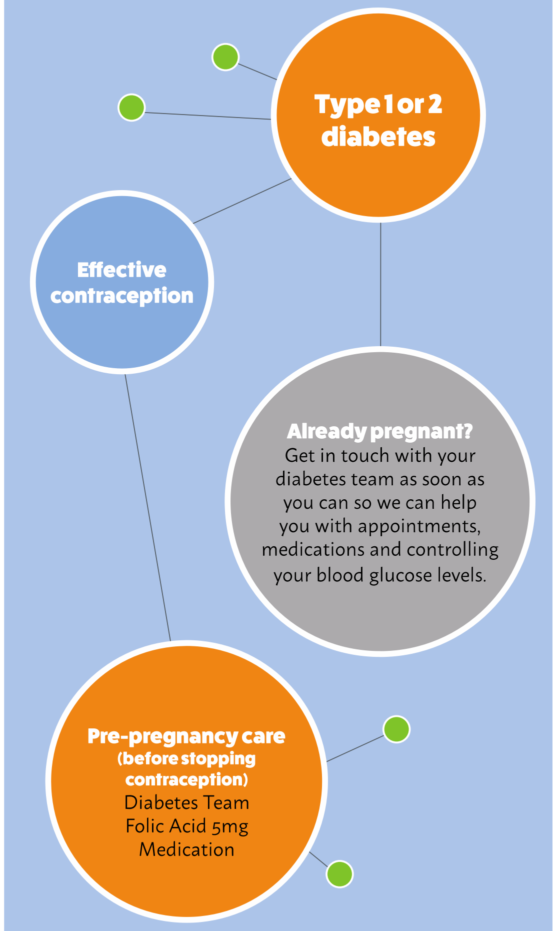

You can reduce your risk of complications during pregnancy

1. Plan ahead
Make an appointment with your diabetes team if you are thinking of becoming pregnant and they will work with you to help you be the healthiest you can be.

2. Control your blood glucose levels
Try and get your HbA1c as close to 48 mmol/mol or 6.5% as you can safely get it (without hypoglycaemia). Test your blood glucose levels before and 1 hour after meals, and before bed.

3. Start taking 5mg Folic Acid tablets daily
Start before conception to help prevent birth defects (especially heart and spine). You will need a prescription for this from your GP.

4. Have your medications reviewed
Particularly blood pressure, cholesterol and diabetes tablets as not all are safe in early pregnancy. Your doctor will help you decide which ones to continue and which to stop or swap to a safer alternative.

5. See a specialist dietician
Discuss pregnancy nutrition with a dietician to help you and your baby to have the best possible health.

6. Ask for help to stop smoking
If you are a smoker a stop-smoking specialist can help you, refer yourself, or ask the diabetes team about a referral.

ESM Figure 2: Electronic preconception care template

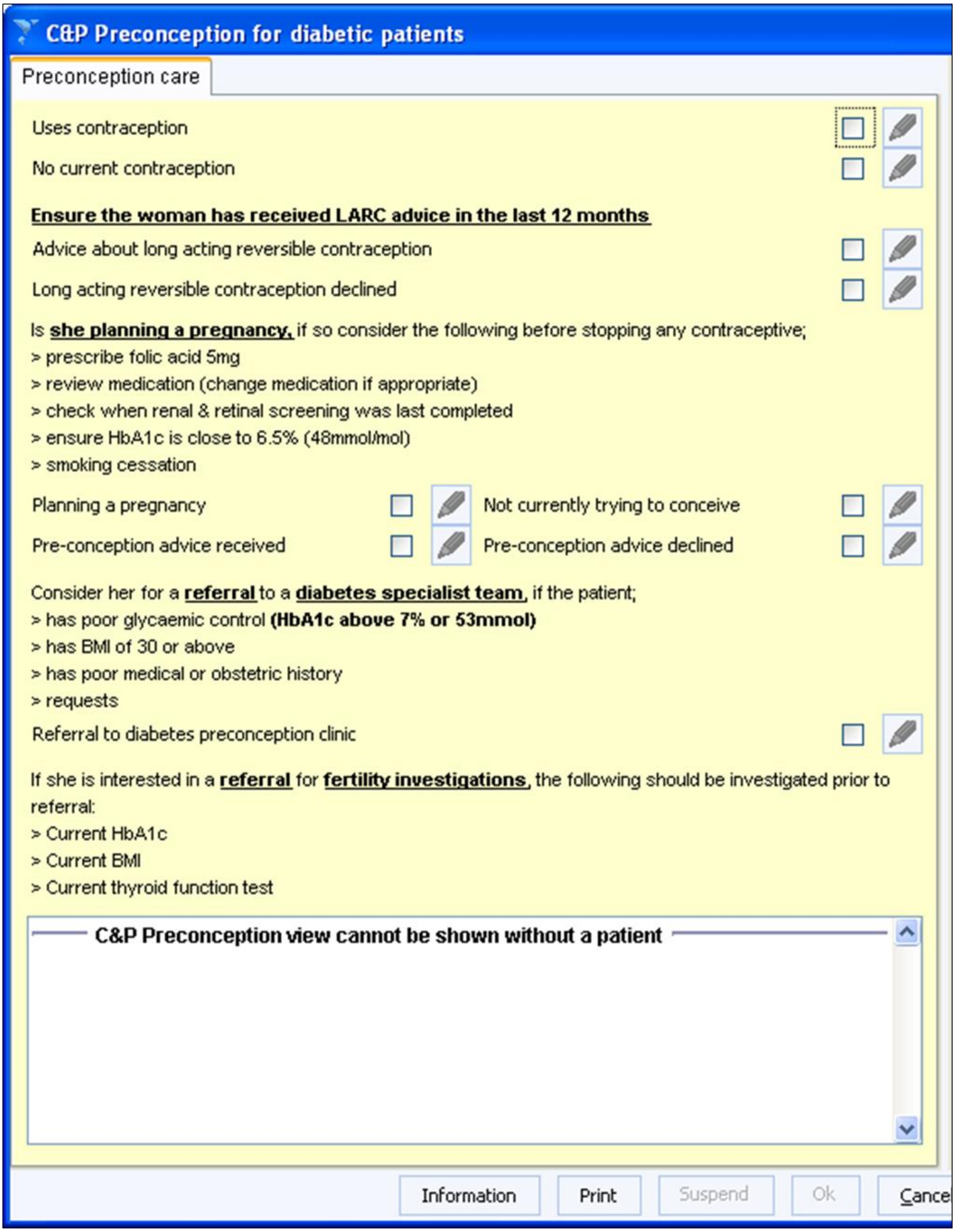

C&P Preconception for diabetic patients

Preconception care

Uses contraception

No current contraception

Ensure the woman has received LARC advice in the last 12 months

Advice about long acting reversible contraception

Long acting reversible contraception declined

Is **she planning a pregnancy,** if so consider the following before stopping any contraceptive;

> prescribe folic acid 5mg

> review medication (change medication if appropriate)

> check when renal & retinal screening was last completed

> ensure HbA1c is close to 6.5% (48mmol/mol)

> smoking cessation

Planning a pregnancy | Not currently trying to conceive

Pre-conception advice received | Pre-conception advice declined

Consider her for a **referral** to a **diabetes specialist team,** if the patient;

> has poor glycaemic control **(HbA1c above 7% or 53mmol)**

> has BMI of 30 or above

> has poor medical or obstetric history

> requests

Referral to diabetes preconception clinic

If she is interested in a **referral** for **fertility investigations,** the following should be investigated prior to referral:

> Current HbA1c

> Current BMI

> Current thyroid function test

C&P Preconception view cannot be shown without a patient

Information | Print | Suspend | Ok | Cance
